# A Robust Method for Ego-Motion Estimation in Urban Environment Using Stereo Camera

**DOI:** 10.3390/s16101704

**Published:** 2016-10-17

**Authors:** Wenyan Ci, Yingping Huang

**Affiliations:** 1 School of Optical-Electrical and Computer Engineering, University of Shanghai for Science & Technology, Shanghai 200093, China; wenyantz@163.com; 2 School of Electric Power Engineering, Nanjing Normal University Taizhou Colledge, Taizhou 225300, China

**Keywords:** visual odometry, ego-motion, stereovision, optical flow, RANSAC algorithm, space position constraint

## Abstract

Visual odometry estimates the ego-motion of an agent (e.g., vehicle and robot) using image information and is a key component for autonomous vehicles and robotics. This paper proposes a robust and precise method for estimating the 6-DoF ego-motion, using a stereo rig with optical flow analysis. An objective function fitted with a set of feature points is created by establishing the mathematical relationship between optical flow, depth and camera ego-motion parameters through the camera’s 3-dimensional motion and planar imaging model. Accordingly, the six motion parameters are computed by minimizing the objective function, using the iterative Levenberg–Marquard method. One of key points for visual odometry is that the feature points selected for the computation should contain inliers as much as possible. In this work, the feature points and their optical flows are initially detected by using the Kanade–Lucas–Tomasi (KLT) algorithm. A circle matching is followed to remove the outliers caused by the mismatching of the KLT algorithm. A space position constraint is imposed to filter out the moving points from the point set detected by the KLT algorithm. The Random Sample Consensus (RANSAC) algorithm is employed to further refine the feature point set, i.e., to eliminate the effects of outliers. The remaining points are tracked to estimate the ego-motion parameters in the subsequent frames. The approach presented here is tested on real traffic videos and the results prove the robustness and precision of the method.

## 1. Introduction

Vehicle ego-motion estimation is a prerequisite for applications such as autonomous navigation and obstacle detection, and therefore acts as a key component for autonomous vehicles and robotics [1]. Conventionally, vehicle ego-motion is measured using a combination of wheeled odometry and inertial sensing. However, this approach has limitations: wheeled odometry is unreliable in slippery terrain and inertial sensors are prone to drift due to error accumulation over a long driving distance, resulting in inaccurate motion estimation. Visual odometry (VO) estimates the ego-motion of an agent (e.g., vehicle and robot) using the input of a single or multiple cameras attached to it [2]. Compared to conventional wheeled odometry, visual odometry promises some advantages in terms of cost, accuracy and reliability. This paper presents a robust vehicle ego-motion estimation approach for urban environments which integrates stereovision with optical flow. 

### 1.1. Related Work 

Visual odometry has been studied for both monocular and stereo cameras. Nister et al. [3] presented one of the first visual odometry using a monocular camera. They used five consecutively matched points to generate an eigenmatrix, and accordingly solved for translational and rotational parameters with a scale factor. A major issue in monocular VO is the scale ambiguity. In monocular VO, feature points need to be observed in at least three different frames. The transformation between the first two consecutive frames in monocular vision is not fully known (scale is unknown) and is usually set to a predefined value. Ke and Kanade [4] virtually rotate the camera to the downward-looking pose to eliminate the ambiguity between rotational and translational ego-motion parameters and improve the Hessian matrix condition in the direct motion estimation process. 

In comparison, stereovision enforces a baseline distance between the two cameras and can triangulate the feature positions with left and right frames to obtain their 3-dimensional (3D) coordinates, thereby resolving the scale ambiguity problem and leading to more accurate results. A solid foundation of a stereovision based visual odometry was provided in [5]. The presented algorithm is for estimating the robot's position by tracking landmarks in a scene. It proved that a stereovision system provides better estimation results than a monocular system. Since then, many stereo-vision based methods have been developed. The two cruxes of these methods are the way of establishing the objective function, and that the feature points selected for the calculation should contain inliers as much as possible. The approach presented in [6] estimated the motion parameters based on dual number quaternions, and the objective function to be minimized was expressed in 3D space. Ni et al. [7] computed poses using 3D space point correspondences. The transformation between the frames was then estimated by minimizing the 3D Euclidean distance between the corresponding 3D points and the solution was solved by an optimization procedure. The main disadvantage of reconstructing the 3D points and formulating the problem as 3D to 3D pose estimation is the sensitivity to stereo reconstruction errors. Some methods estimate the 6 degrees of freedom (6-DoF) motion and the extrinsic parameters of the camera rig in image plane through trifocal tensor [8] or quadrifocal tensor [9] approaches, which help to eliminate reconstruction errors. 

Many stereo-based methods combine stereo with optical flow to establish an objective function fitted with a set of feature points, and accordingly solve for the motion parameters by minimizing the objective function [10,11,12,13,14,15]. One of the key points for these methods is that the feature points selected for computation should not contain outliers, which can be produced by moving objects, mismatching or other factors. Geiger et al. [10] projected feature points from the previous frame into 3D via triangulation using the calibration parameters of the stereo camera rig, and then re-projected them into the original image. The camera ego-motion was computed by minimizing the sum of re-projection errors. They also placed a standard Kalman filter to refine the results. Talukder et al. [11] initially estimated the motion parameters using all image points, rejected outlier points based on initially estimated motion parameters, and re-estimated the motion parameters with the iterative least mean-square error (LMSE) estimation, using filtered feature points. Kitt et al. [12] performed the Longuet–Higgins equations with a sparse set of feature points with given optical flow and disparity to reject feature points lying on independently moving objects in an iterative manner. Kreso et al. [13] removed outliers with a permissive threshold on the re-projection error with respect to the ground truth motion. Fanfani et al. [14] proposed a stereo visual odometry framework based on motion structure analysis. The approach combines the key-point tracking and matching mechanism with the effective key-frame selection strategy to improve the estimation robustness. The approach introduced in [15] was a stereo-based Parallel Tracking and Mapping (S–PTAM) method for map navigation. The process of the method was composed of two parts: tracking the features and creating a map. The extracted feature descriptors are matched against descriptors of the points stored in the map. The matches are then used to refine the estimated camera pose using an iterative least squares minimization method to minimize re-projection errors. 

Urban traffic situations present difficulties for visual odometry since there are many moving objects in a scene. The point set obtained by the above methods unavoidably contains many moving points when a large area of image is composed of moving objects. To achieve true static feature points, Reference [16,17] proposed that only feature points on the ground surface were selected for ego-motion estimation. However, the methods are comparatively poor in adaptability to the environment because a plenty number of effective ground feature points are hard to be extracted in some situations. He et al. [18] utilized visual-inertial sensors to cope with complex environmental dynamics. The robustness of motion estimation was improved since they proposed a visual sanity check mechanism by comparing visually estimated rotation with measured rotation by a gyroscope. However, this method incorporates the gyroscope and thus is not a purely vision-based approach. To overcome these issues, this paper proposes a novel method for estimating the 6-DoF ego-motion in urban environments by integrating optical flow with stereovision. 

### 1.2. Overview of the Approach

In this work, stereovision and the sparse optical flow tracking method are combined to estimate the motion state of a moving camera. An objective function is created by establishing the mathematical relationship between the optical flow caused by a camera’s motion, the depth and the ego-motion parameters. The motion parameters of the camera are computed by minimizing the objective function using an iterative optimization method.

**Figure 1:**
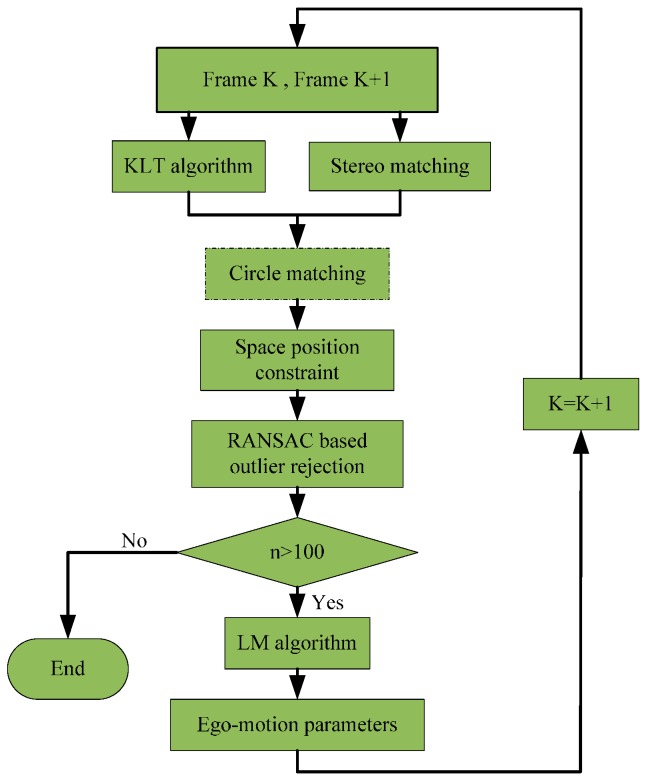
The flowchart of the approach in a tracking cycle.

Figure 1 gives an overview of the approach in a tracking cycle. Firstly, the feature points and their optical flow are detected by using the Kanade–Lucas–Tomasi (KLT) algorithm [19]. The depth information of the feature points is obtained from the stereovision method detailed in our previous work [20]. To remove the outliers caused by KLT mismatching between the consecutive frames and stereo mismatching between left and right images, a circle matching is followed with. Since the circle matching is only conducted in the first frame of a tracking cycle, the block in the flowchart is marked with a dashed frame. The feature points selected by the KLT algorithm may contain a great deal of moving points, and must be filtered out for the minimization computation. To do so, a space position constraint (SPC) is imposed to discriminate between static points and moving points. Afterwards, the Random Sample Consensus (RANSAC) algorithm [21] is employed to further refine the feature point set, i.e., to eliminate the effects of outliers. The remaining feature points are fitted in the objective function and the Levenberg–Marquard (LM) algorithm [22] is used to solve for the six ego-motion parameters by minimizing the objective function. 

After the calculation of the ego-motion parameters of the first frame, the remaining points are tracked in the subsequent frames. In theory, solving for six variables requires a minimum of three known static feature points for computation. In this study, the minimum number of the points involved in the computation is set to be 100 to ensure the computation accuracy. It will start the next round of tracking if the feature points drop below this value as the tracking proceeds. The SPC and RANSAC algorithm are applied in every frame. The combination of the tracking, SPC and RANSAC algorithm increases the proportion of the inliers frame by frame and also reduces computation cost. 

In this work, the stereo matching is employed to measure 3D position (X, Y, Z) of feature points which are used by the objective function and SPC. In addition, the stereo matching is also used in the circle matching process. 

## 2. Proposed Method

### 2.1. 3-Dimensional Motion Model and Objective Function

**Figure 2:**
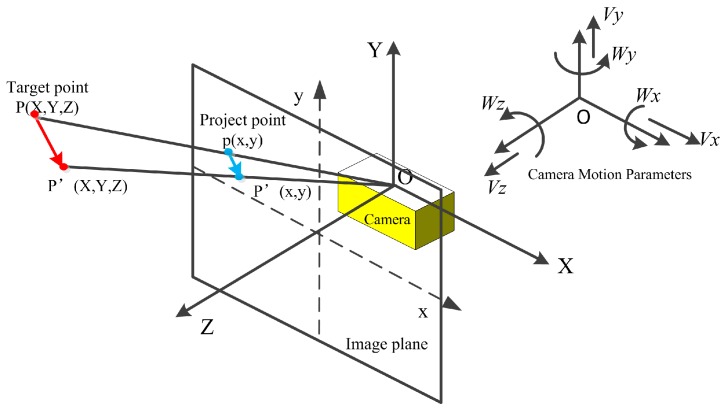
3D motion and planar imaging model.

The camera’s 3-dimensional motion and planar imaging model is represented in Figure 2. The origin of the world coordinate system (X, Y, Z) is located at the center of image coordinates (x, y), and the Z-axis is directed along the optical axis of the camera. The translational velocity of the camera is $\vec{V}=\left(V_{x}{\;V}_{y}{\;V}_{z}\right)$, and the rotational velocity $\vec{W}=\left(W_{x}{\;W}_{y}{\;W}_{z}\right)$.

Assuming a point P(X,Y,Z) in space relatively moves to point $P^{\prime }\left(X,Y,Z\right)$ due to the camera’s movement, the relation between the point motion and camera motion is as below:

(1) $$\frac{\mathrm{dP}}{\mathrm{dt}}=-\left(\vec{V}+\vec{W}\times P\right)$$

The cost product of the point P(X,Y,Z) and the camera’s rotational velocity vector can be represented as:

(2) $$\vec{W}\times P=\left|\begin{array}{ccc} i & j & k\\ W_{x} & W_{y} & W_{z}\\ X & Y & Z \end{array}\right|=\left(W_{y}Z-W_{z}Y\right)i+\left(W_{z}X-W_{x}Z\right)j+\left(W_{x}Y-W_{y}X\right)k$$

where $\left(\begin{array}{ccc} i & j & k \end{array}\right)$ denotes the unit vector in the direction of the X, Y, Z axis, × refers to the cross-product. Thus, Equation (2) can be rewritten as:

(3) $$\vec{W}\times P=\left[\begin{array}{c} W_{y}Z-W_{z}Y\\ W_{z}X-W_{x}Z\\ W_{x}Y-W_{y}X \end{array}\right]$$

The three dimensional velocity $\left(\begin{array}{ccc} \frac{\mathrm{dX}}{\mathrm{dt}} & \frac{\mathrm{dY}}{\mathrm{dt}} & \frac{\mathrm{dZ}}{\mathrm{dt}} \end{array}\right)$ of the point can be obtained as below:

(4) $$\begin{array}{c} \mathrm{dX}/\mathrm{dt}=-\left(V_{x}+W_{y}Z-W_{z}Y\right)\\ \mathrm{dY}/\mathrm{dt}=-\left(V_{y}+W_{z}X-W_{x}Z\right)\\ \mathrm{dZ}/\mathrm{dt}=-\left(V_{z}+W_{x}Y-W_{y}X\right) \end{array}$$

For an ideal pinhole camera model, the image point p(x, y) of the world point P(X, Y, Z) projected in the image plane can be expressed as:

(5) $$x=f\frac{X}{Z},\;y=f\frac{Y}{Z}$$

where f denotes the focal length of the stereo camera. The optical flow $\vec{v}={\left[v_{x}{\;v}_{y}\right]}^{T}$ of P(X,Y,Z) can be obtained by estimating the derivatives along the x-axis and y-axis in 2D image coordinates.

(6) $$\begin{array}{c} v_{x}=\frac{\mathrm{dx}}{\mathrm{dt}}=\frac{1}{Z}\left(f\frac{\mathrm{dX}}{\mathrm{dt}}-x\frac{\mathrm{dZ}}{\mathrm{dt}}\right)\\ v_{y}=\frac{\mathrm{dy}}{\mathrm{dt}}=\frac{1}{Z}\left(f\frac{\mathrm{dY}}{\mathrm{dt}}-y\frac{\mathrm{dZ}}{\mathrm{dt}}\right) \end{array}$$

Integrating Equations (4) to (6) yields the following:

(7) $$\left[\begin{array}{c} v_{x}\\ v_{y} \end{array}\right]=-\left[\begin{array}{cccccc} \frac{f}{Z} & 0 & -\frac{x}{Z} & -\frac{\mathrm{xy}}{f} & \frac{f^{2}+x^{2}}{f} & -y\\ 0 & \frac{f}{Z} & -\frac{y}{Z} & -\frac{f^{2}+y^{2}}{f} & \frac{\mathrm{xy}}{f} & x \end{array}\right]\left[\begin{array}{c} V_{x}\\ V_{y}\\ V_{z}\\ W_{x}\\ W_{y}\\ W_{z} \end{array}\right]=\left[\begin{array}{cc} \frac{M_{V}}{Z} & M_{W} \end{array}\right]\left[\begin{array}{c} \vec{V}\\ \vec{W} \end{array}\right]$$

where $M_{V}=\left[\begin{array}{ccc} -f & 0 & x\\ 0 & -f & y \end{array}\right]$, $M_{W}=\left[\begin{array}{ccc} \frac{\mathrm{xy}}{f} & -\frac{f^{2}+x^{2}}{f} & y\\ \frac{f^{2}+y^{2}}{f} & -\frac{\mathrm{xy}}{f} & -x \end{array}\right]$.

Equation (7) indicates the relationship between optical flow caused by the camera’s ego-motion, depth and six parameters of the camera motion. It is evident that the optical flow and the depth of a minimum of three image points must be known to solve for the six unknown variables, i.e., six camera motion parameters. In this study, the KLT algorithm is used to detect the feature points and to measure their optical flows. The depth is obtained from the stereovision method as reported in our previous work [20]. In Reference [20], a stereovision-based method has been proposed for stereo-matching between left and right images, object segmentation according to position and 3D reconstruction for acquisition of world coordinates. In the stereovision system, the world coordinates are set to be coincided with the coordinates of the left camera.

An objective function is built as:

(8) $$E=\overset{k}{\underset{i=1}{\sum }}\omega_{i}\|\vec{u}-\vec{v}{\|}^{2}$$

where k is the number of the image points, and $\omega_{i}$ is the weight of the point i determined in the following section, $\vec{v}={\left[v_{x}{\;v}_{y}\right]}^{T}$ is the theoretic optical flow in Equation (7), and$\vec{u}={\left[u_{x}{\;u}_{y}\right]}^{T}$ is the optical flow measured by the KLT algorithm. The camera’s motion parameters $\vec{V}$ and $\vec{W}$ can be solved by minimizing Equation (8). The LM algorithm [22] is used to solve the minimization problem. The algorithm is a numerical method to estimate a set of parameters by minimizing an objective function and iteratively refining the parameters. At the first iteration, the LM algorithm works as a gradient method. As it gets near the optimal point, it gradually switches to the Newton-based approach. Good parameter initialization results in a fast and reliable model convergence. In this work, three feature points are initially selected from the point set and put into Equation (8). The computed parameters are taken as the initial value of the LM algorithm. Subsequently, the parameters measured in the current frame are used to initialize the next frame.

### 2.2. KLT Algorithm and Circle Matching

The KLT algorithm [19] is used to select appropriate image points to solve for the minimization problem and to measure their optical flows. The algorithm extracts the Shi–Tomasi corners from an image by computing eigenvalues and selecting the pixels whose minimum eigenvalue is above a certain value. Upon detection of the feature points, the well-known Lucas–Kanade algorithm is used to measure the optical flows of those points. The KLT algorithm also tracks those points. It takes a feature point and its neighborhood as the feature window, and uses the sum of squared difference (SSD) between the frames in the window as the tracking criteria. 

**Figure 3:**
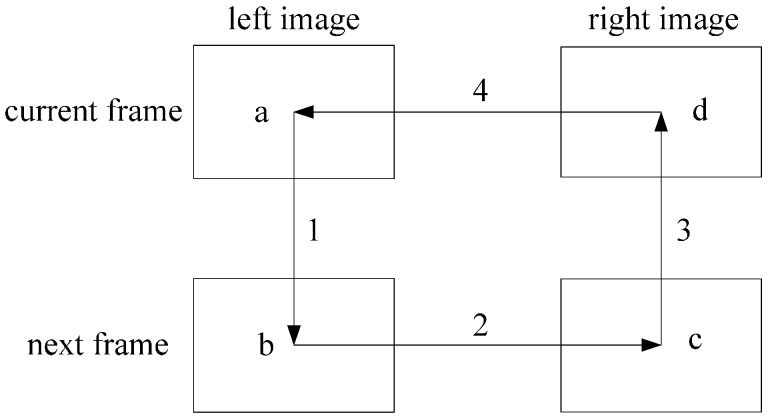
The process of a circle matching.

Following with the KLT detection, a circle matching approach has been adopted to ensure that the feature points are matched correctly between consecutive frames. The process of the circle matching is in the order of a→b→c→d→a as shown in Figure 3. The correspondence between a–b and c–d is established by the KLT algorithm while the correspondence between b–c and d–a is established by the stereo-matching detailed in [20]. A matching is accepted only if the ending feature point is consistent with the starting feature point. The KLT algorithm is normally applied in a monocular imaging sequence. The circle matching mechanism proposed here makes full use of two pairs of image sequences captured from a stereovision rig.

### 2.3. Space Position Constraint 

To obtain correct motion parameters from Equation (8), all feature points used should ideally be selected from the static points such as background and ground. However, the KLT does not discriminate between static points and moving points. Urban traffic scenarios present many moving actors such as vehicles and pedestrians, therefore, the points selected by the KLT contain a great deal of moving points. In consideration of the fact that the movement of a moving point will significantly differ from the one of a static point, a space position constraint is imposed to discriminate them so that moving points will be filtered out. 

The motion of the camera from frame f to f + 1 can be represented by the following 4×4 matrix:

(9) $$M_{f\left(\mathrm{cam}\right)}=\left[\begin{array}{cc} R_{f} & \vec{V_{f}}\\ 0 & 1 \end{array}\right]$$

where $\vec{V_{f}}$ is the translation vector, $R_{f}$ is the rotation matrix given by the Rodriguez’s formula:

(10) $$R_{f}=I+{\left[r\right]}_{\times }\sin\theta +{\left[r\right]}_{\times }^{2}\left(1-\cos\theta \right)$$

where $\theta =\|\vec{W_{f}}\|$, and

(11) $${\left[r\right]}_{\times }=\frac{1}{\theta }\left[\begin{array}{ccc} 0 & -W_{Z\left(f\right)} & W_{Y\left(f\right)}\\ W_{Z\left(f\right)} & 0 & -W_{X\left(f\right)}\\ -W_{Y\left(f\right)} & W_{X\left(f\right)} & 0 \end{array}\right]$$

Assuming that the position of a point P in frame f is $\vec{P_{f}}={\left[\begin{array}{cccc} X_{f} & Y_{f} & Z_{f} & 1 \end{array}\right]}^{T}$, and the position in frame f + 1 is $\vec{P_{f+1}}={\left[\begin{array}{cccc} X_{f+1} & Y_{f+1} & Z_{f+1} & 1 \end{array}\right]}^{T}$, the transform from $\vec{P_{f}}$ to $\vec{P_{f+1}}$ due to the camera motion can be expressed as below if P is an static point:

(12) $$\vec{P_{f+1}}=M_{f}\vec{P_{f}}$$

where $M_{f}$ is the motion matrix from frame f to frame f + 1 of the point P, which can be obtained by inverting the matrix $M_{f\left(\mathrm{cam}\right)}$. Based on the motion smoothness assumption (the frame rate is high enough), the position of point P at frame f+1 can be predicted as ${\vec{P_{f+1}}}_{\mathrm{pre}}=M_{f-1}\vec{P_{f}}$, where $M_{f-1}$ was determined in frame f − 1. It must be noted that the predicted position depends on the camera’s ego-motion and is nothing to do with object’s motion. On other hand, the position of point P at frame f+1, ${\vec{P_{f+1}}}_{\mathrm{mea}}$ can be measured by the stereovision-based method [20]. The difference between the measured position and the predicted position is:

(13) $$\vec{c_{f}}={\vec{P_{f+1}}}_{\mathrm{mea}}-{\vec{P_{f+1}}}_{\mathrm{pre}}$$

For a static point, $\vec{c_{f}}$ should be zero if both measured position and predicted position have no errors. For a moving point, $\vec{c_{f}}$ tends to be large due to its own movement. The maximum acceptable difference between the measured position and the predicted position is given as $\vec{e}={\left[\begin{array}{cccc} \Delta X_{\max} & \Delta Y_{\max} & \Delta Z_{\max} & 1 \end{array}\right]}^{T}$. If the absolute value of any component of $\vec{c_{f}}$ is larger than $\vec{e}$, then the point is discarded. Otherwise the point is assigned a weight as following:

(14) $$\omega =1-\frac{{\left\|\vec{c_{f}}\right\|}^{2}}{{\left\|\vec{e}\right\|}^{2}}$$

which is used in Equation (8). 

Because the position of a feature point in frame f + 1 is predicated from the motion parameters in frame f − 1 (${\vec{P_{f+1}}}_{\mathrm{pre}}=M_{f-1}\vec{P_{f}})$ and the motion parameters in frame f−1 may be slightly different from the one in frame f due to the camera’s acceleration, the basis of setting $\vec{e}$ is the maximum possible position difference of static points caused by the camera’s acceleration between the consecutive frames. In this study, $\vec{e}$ is set to ${\left[\begin{array}{cccc} 0.04 & 0.04 & 0.08 & 1 \end{array}\right]}^{T}$ to ensure a static point will not be wrongly discarded. 

It should be noted that the above setting of $\vec{e}$ does not consider the effect of the error. Actually, the accuracy of the triangulated 3D points decreases with the distance. Consequently, the farther feature points may be wrongly discarded. Thus, a more proper setting of $\vec{e}$ should take errors into consideration by modeling the relationship between the errors and the distance. This will be our future work. 

In theory (without errors), $\vec{c_{f}}$ is actually nothing to do with the distance of a point and only depends on its own movement. In consideration of the fact that that the errors are relatively small compared to the position change of a moving point, this method should not significantly degrade performance for farther points. Our experiments have also verified this.

### 2.4. RANSAC Based Outlier Rejection 

The RANSAC algorithm is employed to further refine the feature point set, i.e., to eliminate the effects of outliers. The RANSAC algorithm [21] is an effective iterative tool to extract the optimal subset from a large data sample to improve the estimation accuracy. A subset S consisting of three points is selected from the points set Q derived from the space position constraint and used to estimate ego-motion parameters using the LM algorithm. The estimated ego-motion parameters are plugged into Equation (7) to compute the optical flow $\vec{v}$ of other points in Q. The points with ${\left\|\vec{u}-\vec{v}\right\|}^{2}$ less than a predefined threshold T are taken as inliers, which constitute a consensus set S* together with the subset S. Repeating this step for M times will generate M consensus set. The six ego-motion parameters are determined by the largest consensus set.

In this work, T is set to 1 pixel based on the experimental empirical value. The number of iterations that is necessary to guarantee a correct solution can be computed as:

(15) $$M=\frac{\log\left(1-p\right)}{\log\left(1-{\left(1-\epsilon \right)}^{s}\right)}$$

where s is the minimum number of data points needed for estimation, p is the required probability of success and ϵ defines the assumed percentage of outliers in the data set.

## 3. Experiments and Results

Experiments have been conducted on the public image database KITTI (Karlsruhe Institute Technology and Toyota Technological Institute) [23]. The KITTI database provides a benchmark platform for evaluation and comparison of different ego-motion estimation approaches. Furthermore, the image sequences are annotated with the ground truth of the ego-motion parameters and the depth.

Two typical urban traffic scenarios with moving objects, are selected as examples to evaluate the approach. The image resolution is 1226 × 370 pixels. The first scenario involves one oncoming bus, and the equipped vehicle moves in a longitudinal direction. In the second scenario, the vehicle is turning right in a bend and the moving object is one car that drives from left to right. 

**Figure 4:**
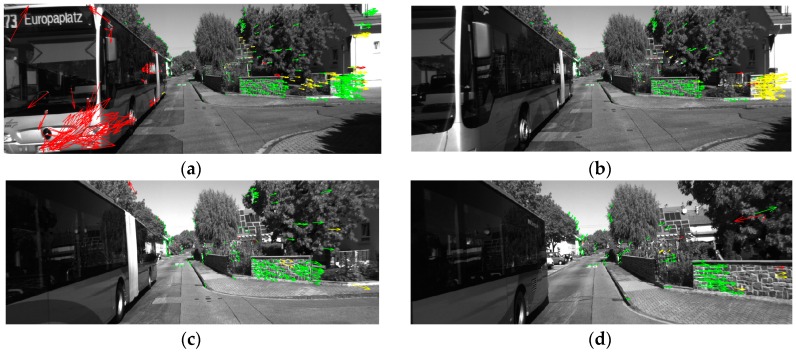
The feature points tracking results in scenario 1. (**a**) Frame 2624; (**b**) Frame 2625; (**c**) Frame 2628; (**d**) Frame 2632. (The green arrows represent the points selected for computation, the red ones are the points rejected by the SPC, and the yellow ones are the points rejected by the RANSAC algorithm.)

Figure 4 shows the results of one tracking cycle (frames 2624–2632) within scenario 1 after the circle matching process. The arrows represent the optical flow of the feature points. The green arrows represent the points selected for computation, the red ones are the points rejected by the SPC, and the yellow ones are the points rejected by the RANSAC algorithm. As shown in Figure 4a, all the moving feature points on the bus have been rejected by the SPC in frame 2624. Table 1 lists the number of the green points and the proportion of the inliers for the four frames. As seen in Table 1, the proportion of the inliers is increasing with the effect of the SPC and RANSAC algorithm. The minimum number of points used for the computation is 104, which is bigger than the threshold of 100. 

**Figure 5:**
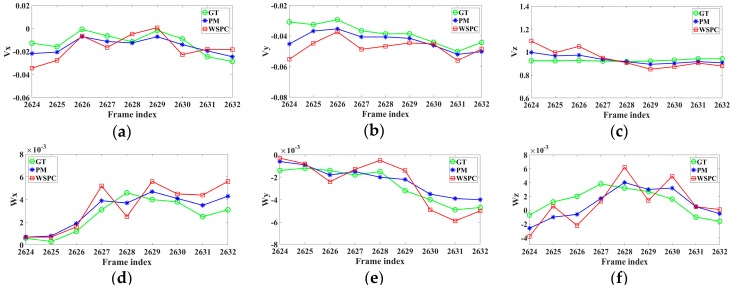
The results of ego-motion parameter estimation in scenario 1. (**a**) $V_{x}$; (**b**) $V_{y}$; (**c**) $V_{z}$; (**d**) $W_{x}$; (**e**) $W_{y}$; (**f**) $W_{z}$.

The ego-motion parameters of frame 2624 to frame 2632 are shown in Figure 5. The parameters are expressed in meters per frame (for translation) and in degrees per frame (for rotation). The results were obtained by the ground truth (GT), the proposed method (PM) and the proposed method without the SPC (WSPC) respectively. It can be seen that the proposed method generates smoother curves than the one without the SPC, and they are closer to the ground truth in the most frames. 

The ego-motion estimation error is expressed by the average translation error (ATE) and the average rotational error (ARE) [23]. The comparison results of PM and WSPC for scenario 1 are shown in Table 2. It can be seen that the PM method is much better than the WSPC method. 

**Figure 6:**
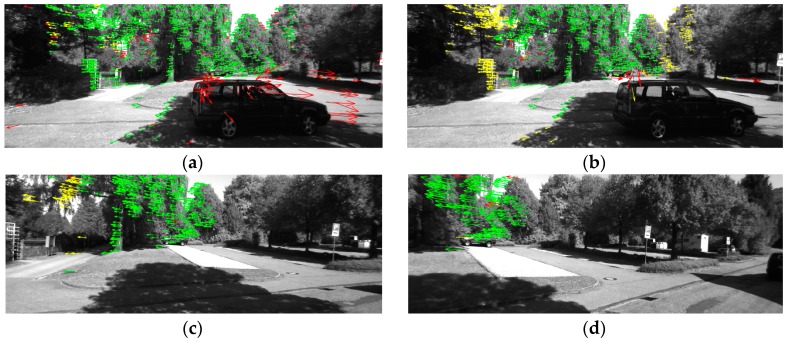
The feature points tracking results in scenario 2. (**a**) Frame 69; (**b**) Frame 70; (**c**) Frame 79; (**d**) Frame 89.

Figure 6 shows the results of one tracking cycle (frames 69–89) within scenario 2 after the circle matching process. As shown in Figure 6a, all the moving feature points on the car have been rejected by the SPC although the equipped vehicle is making a turn. Table 3 lists the number of the green points and the proportion of the inliers. 

**Figure 7:**
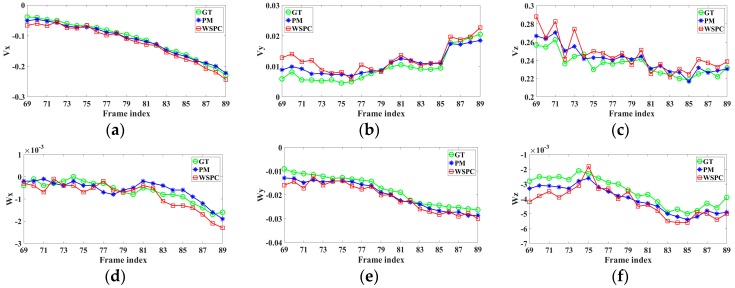
The results of ego-motion parameter estimation in scenario 2. (**a**) $V_{x}$; (**b**) $V_{y}$; (**c**) $V_{z}$; (**d**) $W_{x}$; (**e**) $W_{y}$; (**f**) $W_{z}$.

The ego-motion parameters of frame 69 to frame 89 are shown in Figure 7. For most parameters, the subsequent frames generate more accurate results than the former frames due to the tracking strategy. The comparison of the ego-motion estimation error of PM and WSPC for scenario 2 is shown in Table 4. 

## 4. Evaluation and Comparison

The proposed approach has been compared to the approaches presented in References [10,15]. Reference [10] is known as the VISO2-S method, and Reference [15] is highly ranked as S-PTAM in the KITTI benchmark. The reasons for comparison with these works are: (1) they both are stereovision-based approaches; (2) they both have similar attributes with our method, i.e., solving for the ego-motion parameters by establishing an objective function and accordingly minimizing the objective function using a set of selected feature points.

### 4.1. Robustness

One of the performance measures of a visual odometry is the proportion of the number of inliers $n_{\mathrm{in}}$ accounting for the number of feature points used for the computation $n_{p}$, which can be expressed as the following:

(16) $$P_{\mathrm{in}}=\frac{n_{\mathrm{in}\;}}{n_{p}}\times 100\%$$

If $P_{\mathrm{in}}$ is lower than 20%, ego-motion estimation may fail. In addition, the minimum value of $n_{p}$ must be bigger than a certain number to ensure the computation accuracy. In this study, we set this number to 50. The robustness of an odometry can be evaluated by the ratio of the frames with $P_{\mathrm{in}}>20\%\;{\mathrm{and}\;n}_{p}>50$ to the total frames within an image sequence:

(17) $$P_{s}=\left(\frac{N_{P_{\mathrm{in}\;}>20\%\;\cap \;n_{p}>50}}{N_{f}}\right)\times 100\%$$

where $N_{P_{\mathrm{in}}>20\%\;\cap {\;n}_{p}>50}$ is the number of frames with $P_{\mathrm{in}\;}>20\%$ and $n_{p}>50$, and $N_{f}$ is the total number of frames. The comparison result using 11 KITTI videos is shown in Table 5. 

It can be seen that the proposed method has the greatest robustness. This is mainly because our method integrates the space position constraint together with the RANSAC algorithm into the tracking process to increase the proportion of the inliers and makes sure that the number of feature points used for the computation is bigger than 100.

### 4.2. Absolute Trajectory Error

The absolute trajectory error proposed by Sturm et al. [24] reflects the tracking accuracy, and therefore is used for evaluation and comparison. It is calculated from the root mean squared error (RMSE) of all frames within a trajectory. The comparison result is shown in Table 6. Compared to the mean value of the 11 sequences, our method is better than the VISO2-S method and is slightly worse than the S-PTAM method. It can be noted that our method shows the best performance on sequence 03 and 05, which was collected from urban traffic. All methods have a poor performance on sequence 01, which was collected from a highway scenario with a high driving speed, resulting in a great difference between consecutive frames. The last column of the table lists the average values omitting sequence 01. It can be seen that our method performs best at this time. 

## 5. Conclusions

This paper presents a robust and precise approach for the measurement of 6-DoF ego-motion using binocular cameras. The method integrates stereovision with optical flow to build an objective function fitted with a set of feature points and accordingly solves for the ego-motion parameters by minimizing the objective function. The approach presented here is tested on the KITTI benchmark database and compared with other approaches. The experimental results demonstrate that the approach gives a robustness of 99.31% on outlier removal. The mean of the absolute trajectory error is 25.77 m for all eleven sequences provided by the KITTI. An improvement can be made on setting a more proper threshold $\vec{e}$ of the SPC by modeling the relationship between the position errors and the distance. 

The proposed method follows a traditional procedure, i.e., solving for the ego-motion parameters by establishing an objective function and accordingly minimizing the objective function using a set of selected feature points. However, the contributions of this work can be found as follows: (1) The objective function was built in a unique way. The objective function fitted with a set of feature points is created in the image plane by establishing the mathematical relationship between optical flow, depth and camera ego-motion parameters through the camera’s 3-dimensional motion and planar imaging model. It has significant advantages in avoiding stereo reconstruction errors over the typical 3D to 3D formulation; (2) The combination of the space position constraint with the tracking mechanism and the RANSAC algorithm effectively rejects outliers caused by moving objects and therefore greatly improves the potion of inliers, which makes the approach especially useful in an urban context; (3) The circle matching method fully makes use of two pairs of image sequences captured from a stereovision rig and effectively removes mismatching points caused by the KLT feature detection and stereo matching algorithms. In summary, the proposed approach improves the performance of visual odometry and makes it suitable to be used in an urban environment. 

## Figures and Tables

**Table 1:** Number of the green points and proportion of the inliers.

	Frame 2624	Frame 2625	Frame 2628	Frame 2632
green points	282	200	168	104
proportion of the inliers	91.49%	93.50%	95.24%	95.19%

**Table 2:** Comparison of ego-motion estimation error in scenario 1.

	PM	WSPC
ATE	3.46%	8.51%
ARE	0.0028 deg/m	0.0037 deg/m

**Table 3:** Number of the green points and proportion of the inliers.

	Frame 69	Frame 70	Frame 79	Frame 89
green points	1037	732	372	128
proportion of the inliers	92.57%	94.54%	95.16%	94.53%

**Table 4:** Comparison of ego-motion estimation error for scenario 2.

	PM	WSPC
ATE	3.25%	5.49%
ARE	0.0089 deg/m	0.0151 deg/m

**Table 5:** Comparison of robustness estimation.

	VISO2-S [10]	S-PTAM [15]	Our Method
$P_{s}$	98.27%	98.74%	99.31%

**Table 6:** Absolute trajectory error RMSE in m.

Sequence	VISO2-S [10]	S-PTAM [15]	Our Method
00	29.54	7.66	13.47
01	66.39	203.37	227.51
02	34.41	19.81	11.35
03	1.72	10.13	1.08
04	0.83	1.03	0.96
05	21.62	2.72	1.73
06	11.21	4.10	3.04
07	4.36	1.78	5.84
08	47.84	4.93	9.48
09	89.65	7.15	5.89
10	49.71	1.96	3.16
mean	32.48	24.06	25.77
mean(w/o 01)	29.09	6.13	5.60
